# Physiological effects of KDM5C on neural crest migration and eye formation during vertebrate development

**DOI:** 10.1186/s13072-018-0241-x

**Published:** 2018-12-06

**Authors:** Youni Kim, Youngeun Jeong, Kujin Kwon, Tayaba Ismail, Hyun-Kyung Lee, Chowon Kim, Jeen-Woo Park, Oh-Shin Kwon, Beom-Sik Kang, Dong-Seok Lee, Tae Joo Park, Taejoon Kwon, Hyun-Shik Lee

**Affiliations:** 1 KNU-Center for Nonlinear Dynamics, CMRI, School of Life Sciences, BK21 Plus KNU Creative BioResearch Group, College of Natural Sciences, Kyungpook National University, Daegu, 41566 South Korea; 2 School of Life Sciences, Ulsan National Institute of Science and Technology (UNIST), Ulsan, 44919 South Korea

**Keywords:** Histone demethylase, Neural crest development, Eye formation, Embryogenesis, Organogenesis

## Abstract

**Background:**

Lysine-specific histone demethylase 5C (KDM5C) belongs to the jumonji family of demethylases and is specific for the di- and tri-demethylation of lysine 4 residues on histone 3 (H3K4 me2/3). KDM5C is expressed in the brain and skeletal muscles of humans and is associated with various biologically significant processes. KDM5C is known to be associated with X-linked mental retardation and is also involved in the development of cancer. However, the developmental significance of KDM5C has not been explored yet. In the present study, we investigated the physiological roles of KDM5C during *Xenopus laevis* embryonic development.

**Results:**

Loss-of-function analysis using *kdm5c* antisense morpholino oligonucleotides indicated that *kdm5c* knockdown led to small-sized heads, reduced cartilage size, and malformed eyes (i.e., small-sized and deformed eyes). Molecular analyses of KDM5C functional roles using whole-mount in situ hybridization,* β*-*galactosidase* staining, and reverse transcription-polymerase chain reaction revealed that loss of *kdm5c* resulted in reduced expression levels of neural crest specifiers and genes involved in eye development. Furthermore, transcriptome analysis indicated the significance of KDM5C in morphogenesis and organogenesis.

**Conclusion:**

Our findings indicated that KDM5C is associated with embryonic development and provided additional information regarding the complex and dynamic gene network that regulates neural crest formation and eye development. This study emphasizes the functional significance of KDM5C in *Xenopus* embryogenesis; however, further analysis is needed to explore the interactions of KDM5C with specific developmental genes.

**Electronic supplementary material:**

The online version of this article (10.1186/s13072-018-0241-x) contains supplementary material, which is available to authorized users.

## Background

Embryonic organ development is a highly organized and complex process involving the temporal and spatial expression of genes that control differentiation, maturation, and survival of organs [1]. Additionally, this process involves the formation and migration of cells that are destined to differentiate into specific structures essential for proper development of the organism, such as the neural crest [2]. The neural crest is comprised of stem-like cells that are predestined to migrate extensively and differentiate into specialized cell types during vertebrate embryogenesis [3]. Induction of neural crest cells begins at the gastrula stage of development. Neural crest progenitors are initially identified at the edge of the neural plate, forming a bridge between the neural and non-neural portion of the ectoderm, and require tissue interactions between the neural plate and ectoderm [4]. Before undergoing migration, neural crest cells are localized to the dorsal part of the neural tube [5]. Neural crest cells later migrate throughout the body and give rise to various types of cells, such as melanocytes, craniofacial cartilage and bone, smooth muscles, and peripheral nervous cells [6].

Formation and migration of neural crest cells and development of different organs during vertebrate embryogenesis require regulated gene expression [7, 8], which is affected by the epigenome [9]. Epigenetic modifications, such as methylation, phosphorylation, and ubiquitination, play significant roles in regulating gene expression and interaction to fulfill specific functions [10]; for example, histone lysine methylation leads to the activation or suppression of certain genes [11]. The methylation status of histones is regulated by several types of methyltransferases (KMTs) and demethylases (DMTs) [12]; thus far, two groups of histone demethylases implicated in diverse biological functions have been discovered [13, 14].

Lysine-specific histone demethylase 5C (KDM5C; also known as JARID1C and SMCX) catalyzes the demethylation of lysine 4 on histone 3 (H3K4me3/me2). Since H3K4me3 and H3K4me2 are associated with actively transcribed genes, demethylation of H3K4 by KDM5C causes transcriptional repression [15, 16]. In mammalian cells, KDM5C belongs to a protein subfamily consisting of four members, namely KDM5A/retinoblastoma binding protein 2 (RBP2)/JARID1A, KDM5B/PLU-1/JARID1B, KDM5C/SMCX/JARID1C, and KDM5D/SMCY/JARID1D [17]. KDM5C contains the catalytic jumonji C (JmjC) and jumonji N (JmjN) domains, which are involved in the maintenance of JmjC domain structural integrity and possess an ARID/BRIGHT DNA binding domain [18], a single C5HC2 zinc finger domain located at the C-terminal of the JmjC domain, and two plant homeodomains (PHD) that bind to the methyl lysine residue [19].

The *kdm5c* gene, which is located on the X chromosome, has recently been identified as the gene responsible for X-linked mental retardation (XLMR) [20]. XLMR is a heterogeneous disease that is affected by genetic, environmental, and stochastic factors [21]. Notably, *kdm5c* mutations found in XLMR diminish the ability of KDM5C to demethylate H3K4, indicating that the demethylation activity of KDM5C is critical for brain development [21–23].

KDM5C is highly expressed in the brain and skeletal muscle tissue in humans [21], and human KDM5C is involved in the inhibition of specific neuronal genes. In the mouse brain, *kdm5c* is widely expressed in regions related to cognitive and emotional behaviors, including the prefrontal cortex, hippocampus, and amygdala [24]. *kdm5c*-knockout mice exhibit abnormal social behavior including aggression as well as impaired learning and memory [20, 24]. In addition, KDM5C escapes X-inactivation in both mice and humans [25]. In zebrafish, knockdown of the *kdm5c* homologue induces brain-patterning defects and neuronal cell death, while small interfering RNA (siRNA)-mediated knockdown of *kdm5c* in primary rat granule neurons damaged dendritic morphogenesis [26]. Furthermore, KDM5C has been implicated in renal cancer [27].

In the current study, we investigated the functional significance of KDM5C during *Xenopus* embryogenesis, where the spatiotemporal expression of *kdm5c* indicated that it is a maternal gene. Loss-of-function studies using *kdm5c* morpholino oligonucleotides (MO) demonstrated the significance of this demethylase in neural crest migration and eye development. Whole-mount in situ hybridization (WISH) and reverse transcription-polymerase chain reaction (RT-PCR) analyses indicated that *kdm5c* knockdown led to inhibition of neural crest migration and defects in eye development. Furthermore, transcriptome analysis of *kdm5c* MO-injected embryos showed that KDM5C is critical for morphogenesis of anatomical structures and organogenesis during *Xenopus* embryonic development. Collectively, we concluded that KDM5C plays significant roles in neural crest migration and eye formation during vertebrate development.

## Results

### *kdm5c* is expressed in neural tissues including the neural crest and eyes

**Fig. 1:**
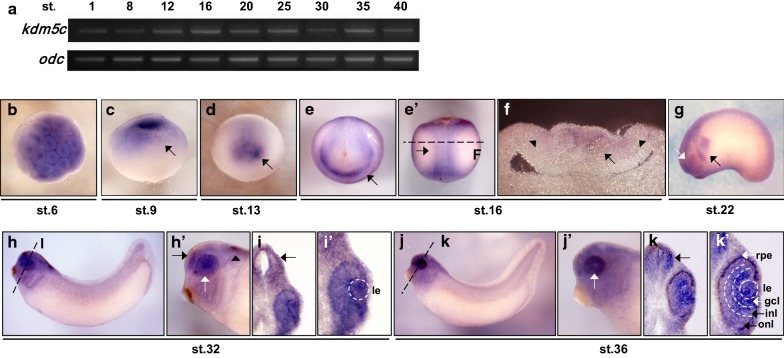
Spatiotemporal expression patterns of *kdm5c* during *Xenopus* embryogenesis. **a** Temporal expression patterns of *kdm5c* analyzed by reverse transcription-polymerase chain reaction (RT-PCR). *kdm5c* was strongly expressed throughout all developmental stages from the one-cell stage to the tadpole development of embryos. Ornithine decarboxylase (*odc*) served as a loading control. **b**–**k′** Spatial expression patterns determined by whole-mount in situ hybridization (WISH). **b** *Xenopus* embryos were collected at developmental stage 6 (cleavage stage) showing localization of *kdm5c* in the animal hemisphere of embryos. **c** *kdm5c* expression at the late blastula stage (st. 9) showing elevated expression levels of *kdm5c* in the animal pole (black arrow). **d** Anterior view of early neurula stage (st. 13) embryos. The expression of *kdm5c* in the prospective early eye field region is indicated by a black arrow. **e** *kdm5c* expression at stage 16 of developing *Xenopus* embryos revealing localization of *kdm5c* in the anterior neural tissue as indicated by a black arrow. **e′** The dorsal view of neurula stage embryos exhibited *kdm5c* expression in the neural plate and neural plate border region and is indicated with a black arrow. **f** Vibratome section of stage 16 embryos showing expression of *kdm5c* in the neural plate and neural plate border regions. **g** Lateral view of early tailbud stage embryos (st. 22) indicating *kdm5c* expression in branchial arches with a black arrow and in the prospective eye regions with a white arrow. **h** Lateral view of tailbud stage (st. 32) embryos showing *kdm5c* expression in the brain of developing embryos. **h′** Detailed view of the tailbud stage embryo in (**h**) showing *kdm5c* expression in the forebrain (black arrow), hindbrain (black arrowhead), and eye (white arrow). **i** Transverse section of the *Xenopus* embryo in (**h**) showing *kdm5c* expression in the midbrain (black arrows). **i′** Detailed view of the embryo in (**i**) showing *kdm5c* expression in the retina and lens (le). **j** Lateral view of late tailbud stage (st. 36) embryos with *kdm5c* expression in the anterior regions including brain and eyes. **j′** High-resolution view of the embryo in (**j**) revealing *kdm5c* expression in the retina (white arrow). **k** Transverse section of the embryo in (**j**) showing *kdm5c* expression in the midbrain regions of late tailbud stage embryos (black arrows). **k′** High-resolution view of the embryo in (**k**) displaying *kdm5c* expression in the lens (le; dotted white circle) as well as outer nuclear layer (onl) and inner nuclear layer (inl; both marked by black arrows). Besides these regions, strong expression of *kdm5c* was detected in the ganglion cell layer (gcl; white arrow) of the eye and retinal pigment epithelium (rpe; white arrow)

To investigate the specific roles of KDM5C during embryogenesis, we first analyzed its gene expression pattern in *Xenopus*. For this purpose, we conducted RT-PCR and WISH analyses. RT-PCR revealed that *kdm5c* is a maternal gene, as it was found expressed throughout embryonic development from the single-cell stage to the tadpole stage (Fig. 1a). The temporal expression pattern of *kdm5c* indicated that this gene possesses essential functions during *Xenopus* development.

WISH analysis was performed to determine the spatial expression patterns of *kdm5c* during *Xenopus* embryonic development at different developmental stages (st. 6, 9, 13, 16, 22, 32, and 36; Fig. 1b–k′). The expression pattern of *kdm5c* indicated that this gene is expressed in the animal hemisphere of developing embryos at developmental stage 6 (Fig. 1b). Additionally, *kdm5c* expression was observed in late blastula-stage embryos showing enhanced expression in the animal pole (st. 9; Fig. 1c). Tissue-specific expression of *kdm5c* was observed during the neurula stage of embryonic development and was found expressed in the early eye field region at stage 13 (Fig. 1d). We also observed *kdm5c* expression in the anterior neural tissues of neurula stage embryos (st. 16; Fig. 1e) with dorsal expression in the neural plate border region (Fig. 1e′). Vibratome section analysis confirmed *kdm5c* expression in neural plate and neural plate border regions (Fig. 1f). We also examined the expression patterns of *kdm5c* during early and late tailbud stages by focusing on the lateral views of developing embryos and by transverse sectioning these embryos for detailed analysis (st. 22, 32, and 36). Our data demonstrated *kdm5c* expression in the branchial arches and eyes of early tailbud stage (Fig. 1g) as well as in the whole brain of late tailbud stage *Xenopus* embryos (Fig. 1h, h′). Although *kdm5c* expression was observed in the whole brain, elevated expression levels of *kdm5c* were detected in the forebrain and hindbrain regions of developing embryos (Fig. 1h′) as well as in the midbrain regions as revealed by transverse embryo sections (Fig. 1i, k). In addition to the whole brain, enhanced *kdm5c* expression was also observed in the retina and lens of *Xenopus* embryos (Fig. 1h′, j′). A detailed view of the embryos through vibratome transverse sections indicated that *kdm5c* is predominantly expressed in the lens and retina (Fig. 1i, k); furthermore, *kdm5c* was found strongly expressed in the ganglion cell layer of the eye (Fig. 1k′). Based on these findings, it is evident that KDM5C is significant during embryogenesis.

### Knockdown of *kdm5c* leads to small-sized head and reduced cartilage size

**Fig. 2:**
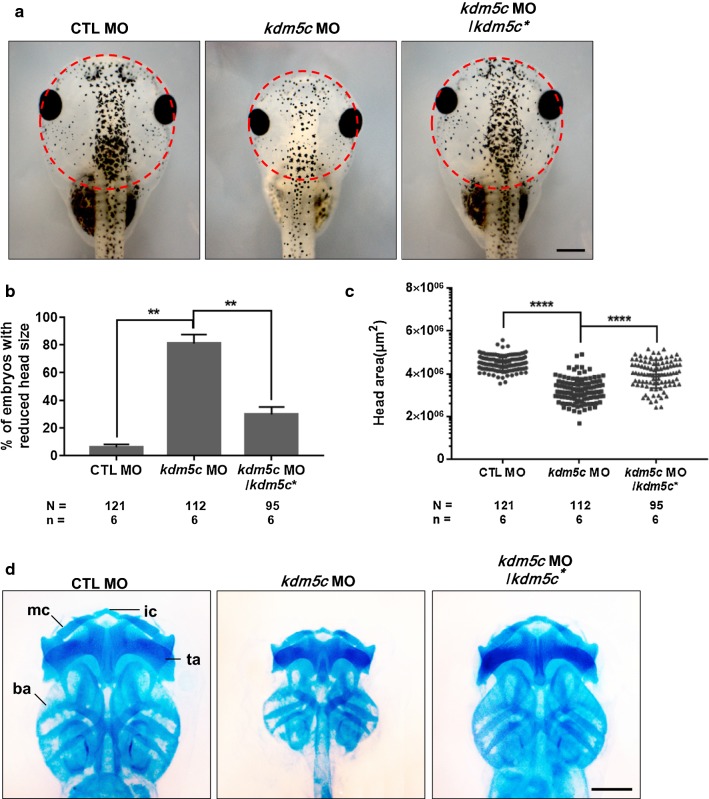
Knockdown of *kdm5c* induced phenotypic malformations. **a** *kdm5c* morpholino oligonucleotide (MO; 48 ng) was injected into embryos at the one-cell stage, and then, embryos were fixed at stage 46. *kdm5c*-depleted embryos exhibited a reduction in head size area (indicated by the dotted red circle) compared with that of control embryos. Small-sized heads of *kdm5*-morphant embryos were effectively rescued by injecting mutant *kdm5c* (kdm5c*) along with *kdm5c* MO. Scale bar = 500 µm. **b** Graphical representation of embryos with reduced head formation compared with control. More than 80% of *kdm5c* MO-injected embryos exhibited small-sized heads. Small-sized heads were effectively rescued by coinjection with kdm5c* RNA. **c** A graph showing the reduction in head area in *kdm5c* morphants. Compared with the control embryos, *kdm5c*-morphant embryos showed approximately 70% reduction in head area, and this phenotypic abnormality was rescued in approximately 88% of embryos coinjected with *kdm5c* MO and kdm5c* RNA. **d** Analysis of cartilage formation in *kdm5c* morphants was performed by fixing the embryos at stage 46 and then staining with alcian blue. Alcian blue staining of *kdm5c* MO-injected embryos demonstrated reduced cartilage size compared with that of control embryos. This phenotypic anomaly was efficiently recovered by rescue experiments. Scale bar = 500 µm. **P < 0.01, ****P < 0.0001; nonparametric, one-tailed Mann–Whitney rank-sum test; ba, branchial arches; CTL, control; ta, tectum anterious; mc, Meckel’s cartilage; ic, infrarostral cartilage

To gain insights into the physiological functions of KDM5C during *Xenopus* embryogenesis, we conducted knockdown studies using *kdm5c* MOs by microinjecting *kdm5c* MO (48 ng) into one-cell stage embryos. To investigate the specificity of *kdm5c* MOs in the knockdown of *kdm5c*, analyzing endogenous KDM5C levels by using anti-KDM5C antibodies is the most suitable; however, due to the lack in availability of anti-KDM5C antibodies for *Xenopus*, we synthesized *kdm5c* mutant RNA using wobble base pairing (kdm5c**) and carried out western blot analysis of control embryos, embryos injected with MO-bound *kdm5c* mRNA, kdm5c*, and kdm5c* together with the MO. Our results revealed that *kdm5c* translation was blocked in MO-bound *kdm5c* mRNA (Additional file 1: Fig. S1). Moreover, KDM5C protein expression of embryos injected with mutated *kdm5c* or coinjected with mutated *kdm5c* and MO verified the specificity of the *kdm5c* MO (Additional file 1: Fig. S1). Microinjection of *kdm5c* MO resulted in phenotypic abnormalities, such as small-sized heads and reduced cartilage size (Fig. 2a–d). Compared with control embryos, over 80% of *kdm5c* MO-injected embryos exhibited smaller-sized heads (Fig. 2b). Moreover, we investigated head size by measuring the head area of *kdm5c* morphants relative to the head area of control MO-injected embryos and observed significantly smaller head areas of approximately 70% upon *kdm5c* depletion (Fig. 2c). To further investigate these cartilage defects, we carried out alcian blue staining of *kdm5c* MO-injected embryos (st. 46). The results indicated that *kdm5c* morphants exhibited a marked reduction in cartilage size compared with that of control MO-injected embryos, whereas cartilage structure was not affected (Fig. 2d).

To rule out the unspecific side effects of MOs using *kdm5c* RNA, we carried out rescue experiments by microinjecting *Xenopus* embryos with *kdm5c* mutant RNA along with *kdm5c* MO. Injection of mutant kdm5c* RNA (1.6 ng) rescued all phenotypic malformations induced by the *kdm5c* MO (Fig. 2a–d); embryos injected with mutant kdm5c* recovered approximately 88% of head area reduction (Fig. 2c). Taken together, these findings indicate that KDM5C is specifically involved in head and cartilage development during embryogenesis.

### KDM5C regulates apoptosis and cell proliferation

**Fig. 3:**
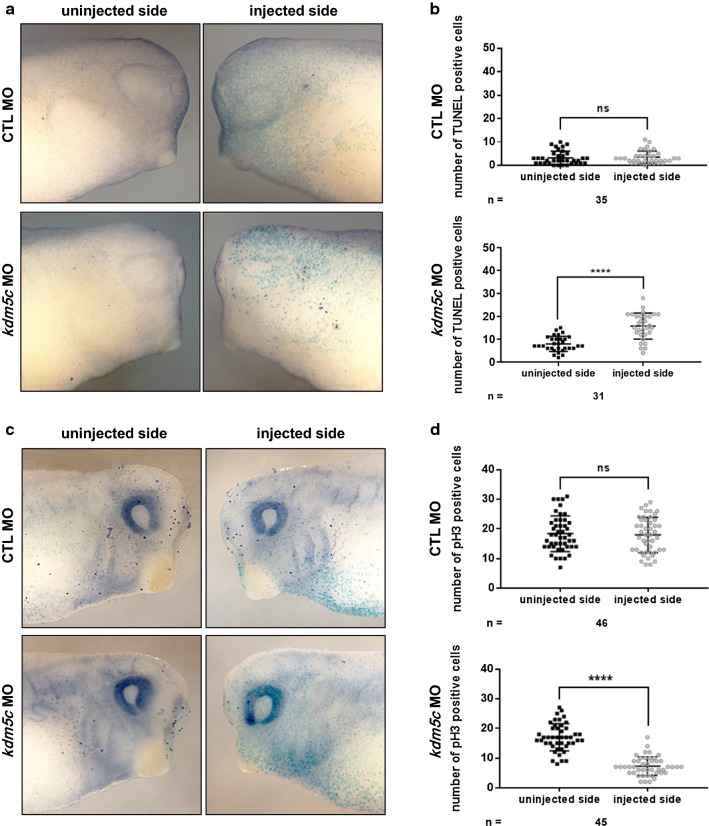
TUNEL and pH3 staining of *kdm5c*-depleted embryos at late tailbud stage (st. 32) of embryonic development. **a** *kdm5c* MO leads to an increase in TUNEL-positive cells on the injected side of embryos compared with the uninjected side. No increase is observed in control MO-injected embryos. **b** Statistical analysis of *kdm5c*-depleted embryos and control embryos showing a significant increase in TUNEL-positive cells in the *kdm5c* MO-injected side compared with uninjected side. **c** Depletion of *kdm5c* results in significant reduction in cell proliferation indicated by pH3-positive cells in the injected side of the embryos compared with the uninjected side. **d** Statistical quantification revealing marked reduction in pH3-positive cells in the *kdm5c* MO-injected side compared with the uninjected side of the embryos. No significant decrease was observed between control embryos. ns, not significant; ****P < 0.0001. CTL, control

Cell number plays a significant role in determining organ as well as whole organism size. To maintain constant size, cell number is tightly controlled by different mechanisms including apoptosis and cell proliferation, which are indispensable for regulating cell number and consequently organ size [28]. To elucidate whether the reduced head and cartilage sizes induced by *kdm5c* knockdown were due to perturbation of apoptosis and cell proliferation, we coinjected *kdm5c* MO and *β*-*galactosidase* mRNA unilaterally into one blastomere of two-cell stage embryos and performed terminal deoxynucleotidyl transferase-mediated dUTP nick end labeling (TUNEL) and pH3 (phospho-histone H3) staining at stage 32 (Fig. 3). The uninjected side of the embryos served as an internal control, while *β*-*galactosidase* mRNA was used as a lineage tracer.

TUNEL staining revealed a significant increase in TUNEL-positive cells after *kdm5c* depletion in the *kdm5c* MO-injected side compared with the uninjected side of the embryos (Fig. 3a, b), indicating involvement of KDM5C in apoptosis regulation. Moreover, pH3 staining indicated significant reduction in cell proliferation in the *kdm5c* MO-injected side of the embryos compared with that of the uninjected side (Fig. 3c, d). Therefore, the mechanism underlying reduced head and cartilage sizes may be the result of a significant increase in apoptosis and marked decrease in cell proliferation due to *kdm5c* depletion.

### Loss of *kdm5c* affects migration and differentiation of neural crest cells

**Fig. 4:**
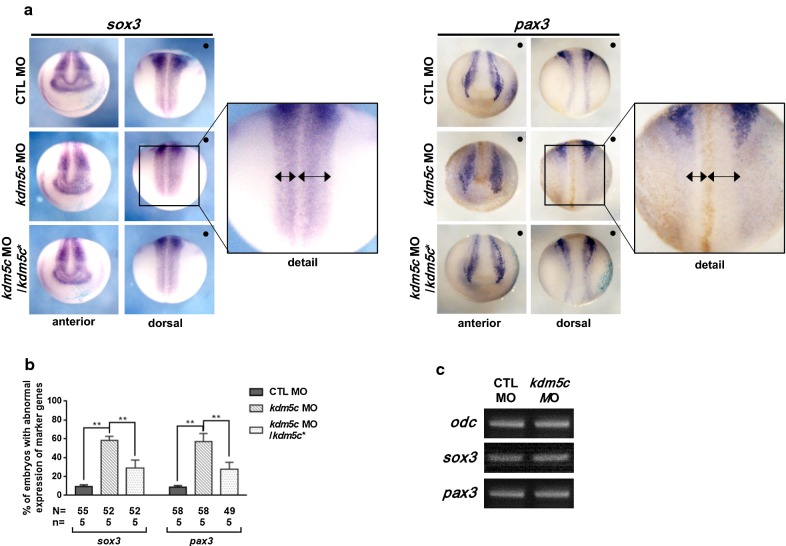
Loss of *kdm5c* influenced the premigratory neural crest cells. **a** *kdm5c* MO (25 ng) was coinjected with *β*-*galactosidase* mRNA into one blastomere of two-cell stage embryos, and then, embryos were fixed at the late neurula stage (st. 16). *β*-*galactosidase* staining indicates the injected side of the embryos. Expansion in neural plate is observed as indicated by *sox3* and *pax3* expressions in the *kdm5c* MO-injected side of the embryos. Black dots (**·**) indicate the injected side of the embryos. Embryos coinjected with *kdm5c* MO and kdm5c* RNA efficiently rescued this expansion in neural plate regions. **b** Statistical analysis of the data revealed significant perturbation of *sox3* and *pax3* expression induced by *kdm5c* knockdown. **c** RT-PCR analysis showed that expression levels of *sox3* and *pax3* are the same in both *kdm5c* morphants and control embryos. ns, not significant; **P < 0.01. CTL, control

**Fig. 5:**
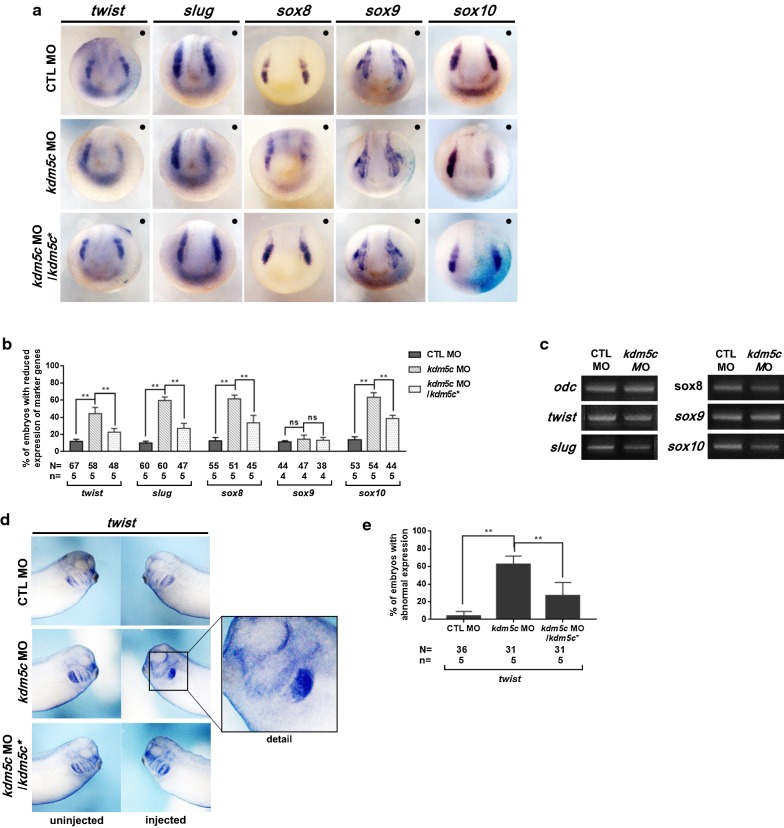
KDM5C is required for neural crest migration. **a** WISH analysis of neural crest markers indicated that the expression of *twist*, *slug*, *sox8*, and *sox10* was significantly reduced on the injected side of the embryos compared with the uninjected side. However, the expression of *sox9* was not affected by *kdm5c* knockdown. The abnormal expression of neural crest specifiers was effectively recovered by rescue experiments. Black dots (**·**) indicate the injected side of the embryos. **b** Statistical analysis of neural crest specifiers expression indicated significant reduction in the levels of all tested markers except for *sox9*, which exhibited no significant change. **c** RT-PCR analysis is consistent with WISH data showing no significant changes in *sox9* expression levels, while all other neural crest specifiers were downregulated. **d** WISH analysis of the neural crest marker *twist* showed expression at stage 32 and the *kdm5c* MO-injected side of the embryos exhibited abnormal neural crest migration compared with that of the uninjected side. Embryos coinjected with *kdm5c* MO and kdm5c* efficiently rescued the abnormal neural crest migration. **e** A graph depicting the significantly perturbed expression levels of *twist* in the *kdm5c* MO-injected side of the embryos compared with the uninjected side. ns, not significant; **P < 0.01. CTL, control

Neural crest development is regulated by the dynamic expression of a number of genes, including *sox3* [29], *pax3* [30], *twist* [31], *slug* [32], and members of the *soxE* family, i.e., *sox8*, *sox9*, and *sox10* [29]. To evaluate the functions of *kdm5c* in neural crest development, we performed a loss-of-function analysis using the *kdm5c* MO and examined its effects on expression of neural crest specifiers. Embryos at the two-cell stage were unilaterally coinjected with *kdm5c* MO and *β*-*galactosidase* mRNA into one blastomere of two-cell stage embryos, after which WISH was performed using these neural crest specifiers. Our results showed *sox3* and *pax3* expressions in the expanded neural plate regions of the *kdm5c* MO-injected side of embryos (Fig. 4a, b), while RT-PCR analysis indicated similar expression levels of *sox3* and *pax3* between control and *kdm5c* MO-injected embryos (Fig. 4c). In contrast to *sox3* and *pax3*, downregulated expression was observed for *twist*, *slug*, *sox8*, and *sox10* in the *kdm5c* MO-injected side; however, *sox9* expression remained unaffected (Fig. 5a, b). *sox8*, *sox9*, and *sox10* belong to the SoxE protein family and play a significant role along with other neural crest specifiers (i.e., *twist* and *snail*). *sox9* is expressed in cranial and cardiac neural crest cells and precedes the expression of *sox8* and *sox10* [33]. Thus, we speculated that the unaffected expression levels of *sox9* in the *kdm5c* MO-injected side are why the cranial cartilage did not exhibit deformations, only a reduction in size (Figs. 2d, 5a, b). To further clarify the expression of these neural crest-specific genes, RT-PCR analysis indicated that expression levels of *twist*, *slug*, *sox8*, and *sox10* were considerably reduced, while *sox9* remained unaltered in *kdm5c* MO-injected embryos compared with control MO (Fig. 5c).

We further examined the *twist* expression pattern during the late tailbud stage (st. 32) to analyze the effect of *kdm5c* knockdown on neural crest migration. Perturbed *twist* expression was observed during later stages of embryonic development after *kdm5c* knockdown, indicating abnormal migration of neural crest cells (Fig. 5d, e). Moreover, the abnormal expression patterns of neural crest specifiers were significantly rescued by injecting *kdm5c* mutant RNA, ruling out any unspecific side effects of the *kdm5c* MOs (Figs. 4a–c, 5a–e). Altogether, these results indicate that KDM5C is required for the expression of neural crest specifiers; thus, perturbation of *kdm5c* expression altered expression patterns and influenced neural crest migration.

### KDM5C is involved in eye development

**Fig. 6:**
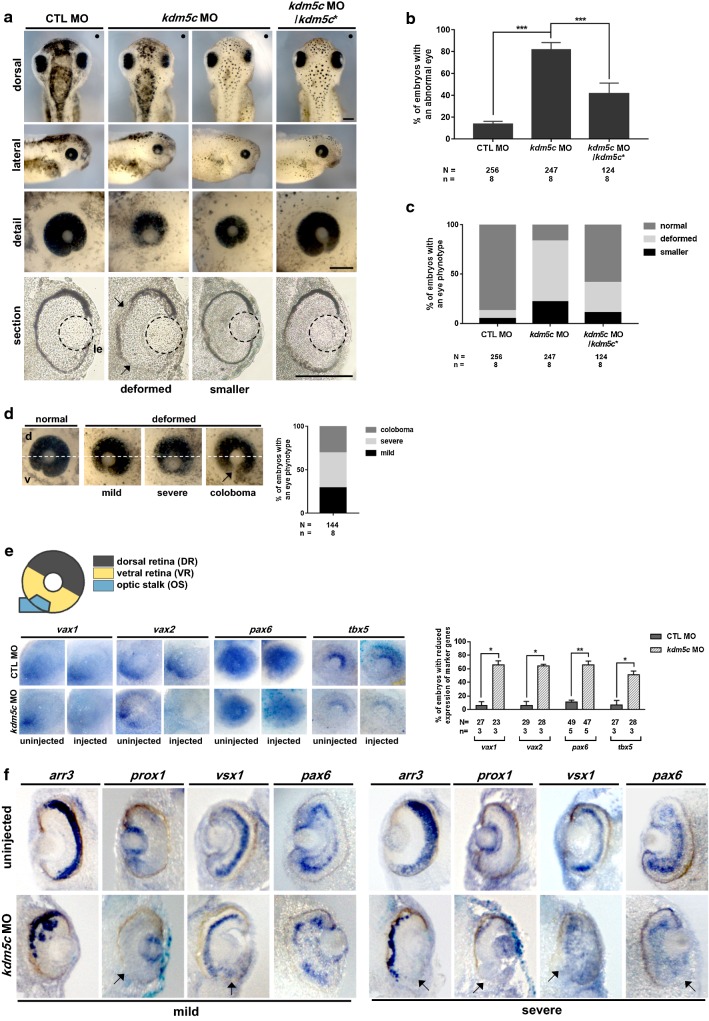
Knockdown of *kdm5c* results in severe eye malformations and affects the expression of eye-specific genes. **a** At stage 40, *kdm5c* morphants exhibited eye defects, such as smaller eyes and optical fissures, compared with that of control embryos. The small and deformed eyes were effectively rescued by coinjection of *kdm5c* MO and kdm5c* RNA. Scale bar = 200 µm. **b** Statistical analysis of embryos with abnormal eyes revealed that more than 80% of *kdm5c* morphants exhibited abnormal eyes compared with that of control embryos. Rescue experiments effectively recovered the eye abnormalities. **c** Percentage of embryos with eye phenotypes, showing *kdm5c* morphant embryos with small eyes (21.75%) and deformed eyes (61.64%). Rescue experiments significantly recovered these eye defects, where only 11.05% of embryos had smaller eyes and 30.28% exhibited deformed eyes. **d** *kdm5c* morphants suffered from colobomas. The mild and severe phenotypes are shown along with statistical quantification. **e** WISH analysis of *kdm5c*-deficient embryos using dorsoventral patterning markers of the retina. *vax1*, *vax2*, *pax6*, and *tbx5* expressions were significantly reduced on the injected side compared with the uninjected side. No significant change in marker expression was observed for control embryos. Statistical analysis of the data is provided. **f** Vibratome section analysis of embryos stained with retinal cell-specific markers (*arr3*, *prox1*, *vsx1*, and *pax6*). Perturbed expression of all tested marker genes indicated disturbed ganglion cell layers as well as retinal lamination defects. The mild and severe phenotypes are provided for all markers. *P < 0.05; **P < 0.01; ***P < 0.001. CTL, control

As our spatial expression analysis of *kdm5c* in *Xenopus* embryos indicated enriched expression of *kdm5c* in the eye regions (Fig. 1g–k′) and on the basis of the well-established *Xenopus* fate maps, we sought to investigate the involvement of *kdm5c* in eye development during *Xenopus* embryogenesis. Thus, we conducted unilateral microinjection of *kdm5c* into eight-cell stage *Xenopus* embryos and found that *kdm5c* morphants exhibited significantly smaller and deformed eyes, i.e., coloboma/optic fissures, compared with that of control embryos (Fig. 6a, d). Statistical analysis revealed that compared with control embryos, more than 80% of *kdm5c* MO-injected embryos exhibited eye defects (Fig. 6b) and among the *kdm5c* morphants, approximately 20% exhibited small-sized eyes and 60% possessed deformed eyes (Fig. 6c). Additionally, histological analysis of the eye structure through vibratome sections indicated that *kdm5c* morphants exhibited abnormal retinal pigment epithelium (Fig. 6a). For validating the specificity of *kdm5c* MO-induced eye defects, we performed rescue experiments that confirmed the eye malformations observed in *kdm5c* morphants were specifically caused by a depletion of *kdm5c* and not through unspecific side effects of *kdm5c* MOs (Fig. 6a–c). In short, our results implicate *kdm5c* in eye development during *Xenopus* embryogenesis.

Loss of *kdm5c* induced phenotypic eye defects of coloboma/optic fissures in the morphant embryos (Fig. 6a–d), which may have resulted from non-closure of the choroid fissure, leading to coloboma. Dorsoventral (DV) patterning of the retina is important for the choroid fissure, and impairment of DV patterning can result in colobomas. Retina DV patterning is controlled by asymmetric expression of transcription factors, such as *vax1* (optical stalk-specific), *vax2* (optical stalk and ventral retina-specific), *pax6* (ventral and dorsal retina-specific), and *tbx5* (dorsal retina-specific), that regionalize optic vesicle into three compartments, i.e., optic stalk, dorsal retina, and ventral retina [34]. Thus, we examined the effects of *kdm5c* knockdown on DV patterning by analyzing the expression of DV-patterning markers (*vax1*, *vax2*, *pax6*, and *tbx5)* through WISH. We found that depletion of *kdm5c* significantly downregulated *vax1*, *vax2*, *pax6* expressions, while *tbx5* expression was slightly reduced (Fig. 6e); thus, the reduced expression of DV-patterning markers may be responsible for the colobomas observed in *kdm5c* morphants.

In addition to colobomas, vibratome sections of *kdm5c* morphants indicated retinal lamination defects (Fig. 6a). Therefore, we performed WISH with the well-known eye-specific markers *arr3* (photoreceptor cell-specific), *prox1* (horizontal cell-specific), *vsx1* (bipolar cell-specific), and *pax6* (ganglion and amacrine cell-specific) to further analyze *kdm5c* morphant eyes (st. 40). We obtained both mild and severe phenotypes through WISH analysis as well as severe disorganization of retinal cell layers (Fig. 6f). Overall, our findings indicate that *kdm5c* knockdown induced severe eye defects, including colobomas and perturbed retinal lamination.

### KDM5C is significant for early eye field induction and differentiation

**Fig. 7:**
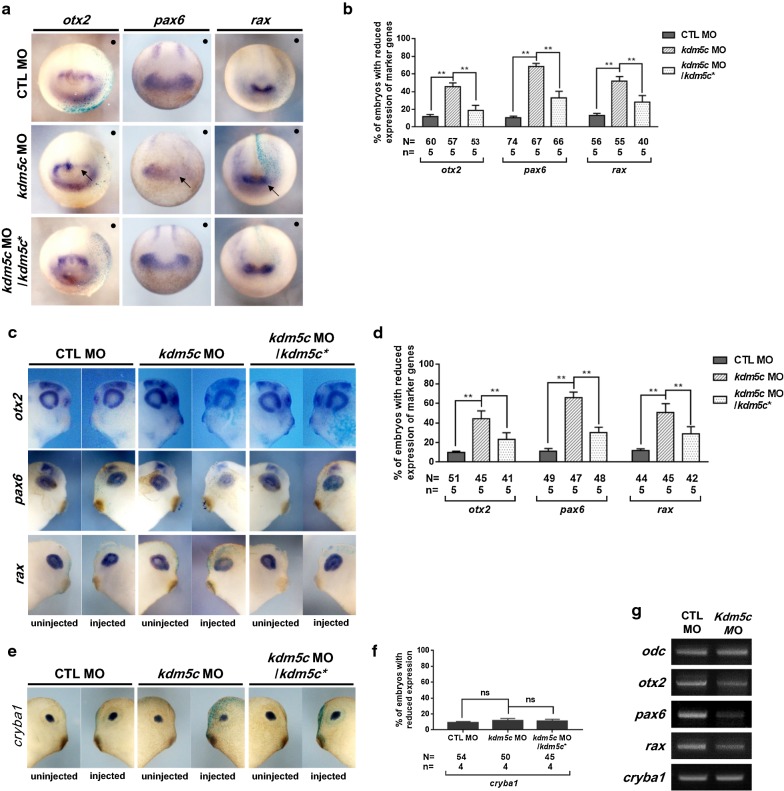
KDM5C is involved in eye field induction and differentiation. **a** *kdm5c* MO (13 ng) was coinjected with *β*-*galactosidase* mRNA into one blastomere of eight-cell stage embryos. Embryos were fixed at the neurula stage (st. 16).* β*-*galactosidase* staining indicates the injected side of the embryos. WISH analysis was performed using *otx2*, *rax*, and *pax6* markers. *otx2*, *rax*, and *pax6* expressions were downregulated on the injected side of the embryos. Changes in expression levels of neural and eye-specific markers were efficiently rescued by coinjecting *kdm5c* MO and kdm5c* RNA. **b** Statistical analysis of embryos exhibiting abnormal expression patterns of eye field induction and differentiation markers compared with that of control embryos. **c** WISH analysis of *otx2*, *rax*, and *pax6* at stage 32 of developing embryos is in agreement with the analysis performed at stage 16 of developing embryos. Downregulated expression on the injected side of the embryos indicated that *kdm5c* is significant for eye field induction and differentiation. **d** Statistical analysis of embryos exhibiting abnormal expression patterns of eye field induction and differentiation markers compared with that of control embryos. **e** WISH analysis of stage 32 embryos using the lens-specific marker *cryba1* indicated that expression of *cryba1* is not affected by *kdm5c* depletion. **f** Percentage of embryos with reduced expression indicated that *cryba1* expression was not affected by *kdm5c* knockdown. **g** RT-PCR analysis using eye field induction and differentiation markers as well as *cryba1* revealed that KDM5C downregulated the expression of *otx2*, *rax*, and *pax6* but not *cryba1*, which remained unaffected. ns, not significant; **P < 0.01; CTL, control

We further investigated the roles of KDM5C at the molecular level during eye development by coinjecting *kdm5c* MO and *β*-*galactosidase* mRNA unilaterally into one dorsal blastomere of eight-cell stage embryos. WISH analysis of these *kdm5c* MO/*β*-*galactosidase* mRNA-coinjected embryos was performed to evaluate the effect of *kdm5c* knockdown on eye field induction and differentiation by examining the expression patterns of *otx2* [35], *rax* [36], and *pax6* [37] at stage 16 of embryogenesis. Compared with control, *otx2, rax*, and *pax6* expressions were downregulated in the *kdm5c* MO-injected side of the embryos (Fig. 7a, b). Furthermore, we examined the effect of *kdm5c* knockdown on eye differentiation at stage 32 (Fig. 7c, d) and found that all tested eye-specific markers exhibited reduced expression levels in the *kdm5c* MO-injected side, whereas normal expression was observed on the uninjected side of the embryos. WISH analysis with *cryba1* specific for the vertebrate eye lens [38] was also conducted at stage 32; however, *kdm5c* knockdown did not affect lens development during *Xenopus* embryogenesis (Fig. 7e, f). RT-PCR analysis further confirmed that the presence of KDM5C is significant during eye field induction and differentiation but is not required during eye lens development (Fig. 7g). Moreover, rescue experiments effectively recovered the reduced expression levels of eye-specific markers induced by *kdm5c* knockdown (Fig. 7a–f), verifying the specificity of KDM5C in eye development during *Xenopus* embryogenesis. Altogether, our results demonstrate that KDM5C plays an important role during eye field induction and differentiation and that loss of *kdm5c* results in anomalies of retina formation during *Xenopus* embryogenesis.

### KDM5C is required for organogenesis and morphogenesis

To pinpoint target genes specifically affected by *kdm5c* knockdown, we performed a transcriptome analysis of *kdm5c* morphants. Total RNA of *kdm5c* morphants was extracted and processed for transcriptome and RNA sequence analysis. RNA sequence analysis identified important gene groups (Additional file 2: Fig. S2); genes were classified into 19 groups using PANTHER gene ontology; and a bar chart was plotted based on the downregulated expression of these gene groups in the *kdm5c* morphants (Additional file 2: Fig. S2). These analyses indicated that *kdm5c* plays significant roles in morphogenesis.

**Fig. 8:**
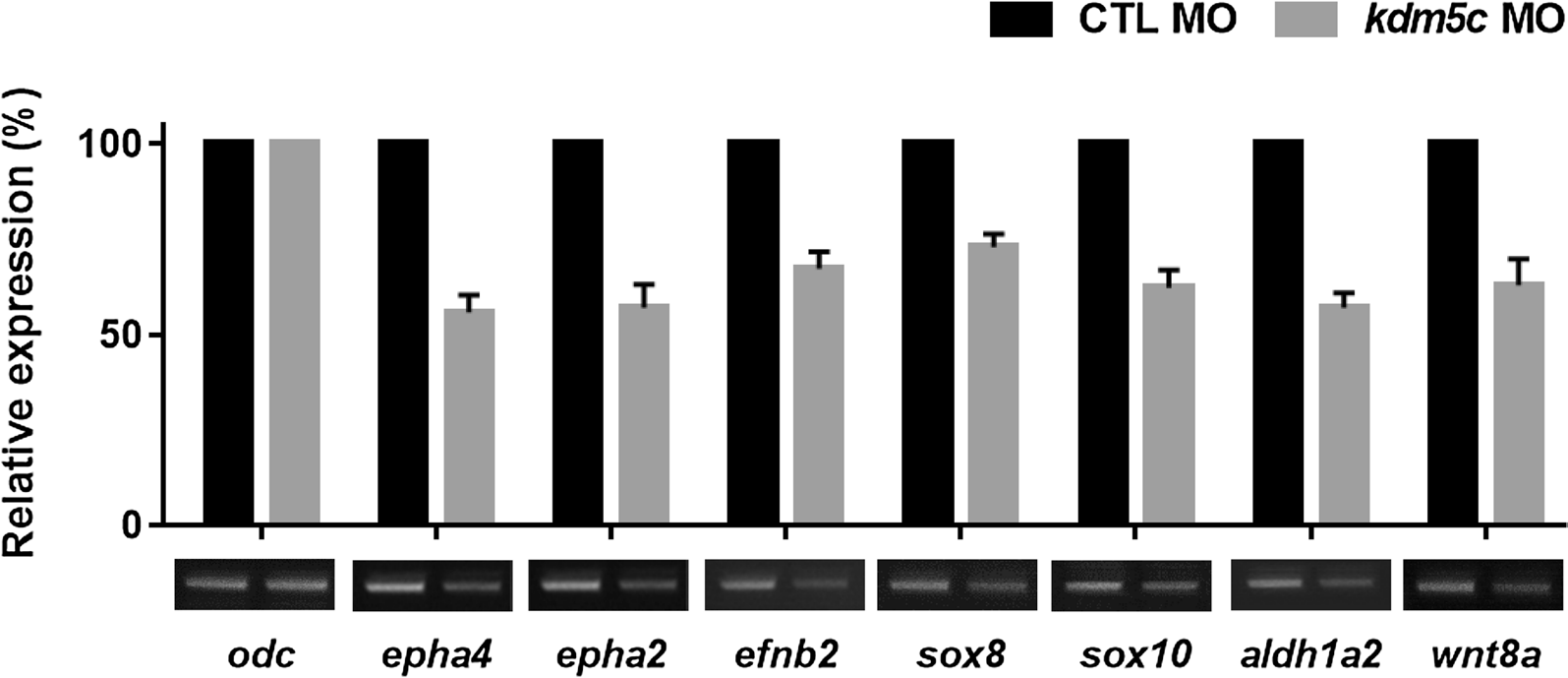
KDM5C plays important roles in morphogenesis and organ development. RT-PCR analysis of genes (with high fold-change values) selected after RNA sequence analysis. Expression levels of *sox8*, *sox10*, and *wnt8a* were significantly reduced in *kdm5c* morphants. Additionally, downregulation of *ephA4, ephA2, efnb2*, and *aldh1a2* (genes associated with eye development) was observed as a result of *kdm5c* knockdown. CTL, control

To validate RNA sequence analysis, genes with high fold-change values were selected and RT-PCR was performed to analyze the expression of these genes in *kdm5c* morphants. *epha4*, *epha2*, *efnb2*, *sox8*, *sox10*, *aldh1a2*, and *wnt8a* are all genes involved in the regulation of eye and neural crest development during embryogenesis [39–43]. We found that *epha4*, *epha2*, *efnb2*, *sox8*, *sox10*, *aldh1a2*, and *wnt8a* were downregulated among several other genes (Fig. 8). RT-PCR showed reduced gene expression of *sox8*, *sox10*, and *wnt8a*, confirming that *kdm5c* is essential for the regulation of neural crest development (Figs. 5c, 8). Moreover, the downregulated expression patterns of *epha4*, *epha2*, *efnb2*, and *aldh1a2* validated the involvement of KDM5C in eye development during embryogenesis (Fig. 8). Overall, our results demonstrate that KDM5C is critical for morphogenesis and specifically influences neural crest development and eye formation during embryonic development.

## Discussion

Members of the jumonji family of histone demethylases have emerged as significant regulators of epigenetic events [16, 44] and have been implicated in diverse biological processes ranging from developmental events to the pathogenesis of several diseases [45]. KDM5C catalyzes the di- and tri-demethylation of H3K4 and is associated with transcriptional repression [15]. This demethylase is strongly expressed in human brain and skeletal muscle tissues and is associated with memory defects and aggressive tendencies in *kdm5c*-knockout mice [25]. KDM5C has also been implicated in XLMR as *kdm5c* mutations are typically observed in XLMR patients [20, 21]. Moreover, KDM5C has been studied with regard to its significance in X-inactivation and has been associated with certain types of cancer [22, 23]. However, KDM5C has not been investigated for its roles during embryonic development. In the current study, we sought to explore the physiological significance of KDM5C during *Xenopus* embryogenesis.

*Xenopus* is an excellent animal model for the study of gene function as genetic overexpression or knockdown can be induced by RNA or DNA microinjection into fertilized eggs; consequently, gene expression can be observed throughout different stages of embryonic development [45]. The spatiotemporal expression pattern of *kdm5c* demonstrated that it is a maternal gene (Fig. 1a) and is specifically expressed in the branchial arches as well as the prospective eye field and brain of developing embryos (Fig. 1b–k′). The functional significance of *kdm5c* was evaluated by microinjecting *kdm5c* MOs into one-cell stage embryos. Loss-of-function analysis revealed that *kdm5c* is essential for proper embryonic development and that loss of *kdm5c* induced phenotypic malformations—reduced head size, smaller cartilage, and defective eyes—in the morphant embryos (Figs. 2, 6). The reduction in cartilage and head size was supported by increased apoptosis and decreased cell proliferation in *kdm5c* morphant embryos (Fig. 3). These findings led us to hypothesize that KDM5C plays critical roles in embryonic morphogenesis and organ development. Our observed phenotypes of reduced head and cartilage size are in agreement with the finding that *kdm5c* mutations lead to XLMR in humans, who also exhibit microcephaly [46]. It would thus be interesting to further examine whether exogenous *kdm5c* can rescue microcephaly in affected humans.

Neural crest cells are multipotent cells that can differentiate into several cell types, such as melanocytes, skeletal muscle, craniofacial cartilage, and bone [47, 48]. Neural crest morphogenesis has three distinct phases and is associated with the differentiation ability of neural crest cells [8]. Transcription factor genes including *sox3* [30]*, pax3* [29]*, sox8* [49], *sox9, sox10* [50], *twist* [31, 51], and *slug* [32] are involved in the migration and differentiation of the neural crest. *kdm5c* knockdown led to reduced head and cartilage sizes; thus, investigating these malformations on a molecular level revealed that KDM5C is involved in the migration and differentiation of neural crest cells by altering expression of neural crest specifiers (Figs. 4, 5).

The process of organogenesis is controlled by several distinct epigenetic events [1]. Our knockdown experiments showed that loss of *kdm5c* induced eye defects such as coloboma and disturbed retinal lamination in the developing embryos. Coloboma formation in *kdm5c* morphants may be the result of perturbed expression of DV-patterning markers (Fig. 6e) and *aldh1a2* (retinoid metabolism-specific; Fig. 8). Retinoic acid plays a significant role in eye development through retinoic acid receptor (RAR) signaling that are involved in regulation of choroid fissure closure [52]. *aldh1a2* is involved in the regulation of RAR signaling [52, 53], and thus, its downregulated expression may be the underlying cause of coloboma in *kdm5c* morphants. WISH analysis using retinal cell-specific markers revealed that *kdm5c* knockdown affected the ganglion cell layer and resulted in malformed retinas. Although the eyes were in the proper position, the retinas of injected embryos were extensively malformed (Fig. 6a, d). Altogether, detailed analysis of eye defects in *kdm5c* MO-injected embryos (Fig. 6) indicated the importance of KDM5C in eye development during *Xenopus* embryogenesis.

To investigate the involvement of KDM5C in early eye field induction and differentiation, we analyzed marker gene expression during early (st. 16) and late (st. 32) stages of embryonic development. We found that *otx2*, *rax*, and *pax6* were strongly inhibited upon *kdm5c* knockdown (Fig. 7a–d). This is consistent with previous observations that perturbation in expression patterns of any of these genes is associated with eye defects [54–56]. Interestingly, these genes are associated with eye abnormalities in humans, which includes small eyes and colobomas [35, 37]. Therefore, it will be interesting to uncover whether KDM5C is involved in human ocular defects.

Morphogenesis is a critical biological process regulated by a set of genes that sequentially turn the developmental process on or off in a precise spatiotemporal pattern [57]. The expression of these regulatory genes is in turn controlled by several upstream factors. RNA sequence analysis of *kdm5c* MO-injected embryos demonstrated the significance of KDM5C in organogenesis and morphogenesis of anatomical structures (Additional file 2: Fig. S2). RT-PCR data further confirmed the downregulated expression patterns of *epha4*, *epha2, efnb2*, and *aldh1a2*, which are all genes associated with eye development, as well as reduced gene expression of *sox8*, *sox10*, and *wnt8a* which are involved in neural crest migration (Fig. 8). Previous studies have shown that *epha4* and its interacting partner *sipa1l3* are significant during eye development and that their depletion led to an increase in the Wnt/β-catenin target *axin2* [58]. As *kdm5c* depletion is associated with downregulation of *epha4* and *wnt8a* (Fig. 8), it is possible that KDM5C is also involved in Wnt signaling. Therefore, further elucidation of Wnt signaling regulation by KDM5C is warranted.

## Conclusion

The association of KDM5C with neural crest specifiers and key genes involved in eye development provides additional information regarding the complex and dynamic genetic networks that regulate neural crest and eye development. Furthermore, our findings highlight the significance of epigenetic regulators in controlling the spatiotemporal expression of genes during embryonic development. Our observations also raise the possibility that this repressive histone marker may contribute to developmental disorders owing to its critical role in regulating methylation patterns of key developmental genes.

## Materials and methods

### Plasmids and reagents

cDNA was synthesized from total RNA of tailbud stage embryos. Based on the *kdm5c* sequences in NCBI and Xenbase, primers were designed for cloning *kdm5c*. Flag-tagged *kdm5c* mRNA was generated by PCR and a plasmid was constructed using the pCS107 vector, which included restriction sites for *Cla*I and *Xho*I.

### *Xenopus* growth conditions and in vitro fertilization

This study was performed in strict accordance with the guidelines of the Animal Care and Use Committee and in agreement with international laws and policies (National Institutes of Health Guide for the Care and Use of Laboratory Animals, Publication No. 85-23, 1985). The Institutional Review Board of the Ulsan National Institute of Science and Technology approved the experimental use of amphibians (Approval No. UNISTACUC-16-14). All members of our laboratory attended educational and training courses on the proper care and use of experimental animals. Adult *Xenopus* obtained from the Korean *Xenopus* Resource Center for Research were housed at 18 °C under 12-h light/12-h dark conditions in containers recommended by the Institutional Review Board of the Ulsan National Institute of Science and Technology. Ovulation was induced in *Xenopus* females by injecting 1000 IU of human chorionic gonadotropin into the dorsal lymph sac in the evening before the experiment. The next day, eggs were collected in 60-mm petri dishes containing 1X MBS (88 mM NaCl, 5 mM HEPES, 2.5 mM NaHCO_3_, 1 mM KCl, 1 mM MgSO_4_, and 0.7 mM CaCl_2_, pH 7.8) by squeezing *Xenopus* females. After several washes with 0.1X MBS, eggs were fertilized using a sperm suspension solution derived from the isolated testes of sacrificed male frogs. After successful fertilization, the jelly coat was removed by swirling the embryos in 2% l-cysteine solution, and then, the embryos were washed five times with 0.5X MBS. Unfertilized eggs and dead embryos were removed, and then, healthy embryos were transferred into 0.5X MBS containing 2% Ficoll^®^ 400 (GE Healthcare, Little Chalfont, UK) at 15–18 °C.

### mRNA synthesis and *Xenopus* embryo microinjection

For microinjection, capped mRNAs were synthesized using the SP6 mMessage mMachine^®^ kit (Ambion, Austin, TX). pCS107/*kdm5c*-Flag constructs were linearized with *Apa*I. The *kdm5c* MO consisted of 25 nucleotides and was designed as follows: 5′-ATGTTGAACATGGAGACTGAAGACT-3′ (Gene Tools, Philomath, OR). mRNA or kdm5c MO were coinjected into one cell stage embryos while for WISH analysis mRNA or *kdm5c* MO were unilaterally injected into two-cell and eight-cell stage embryos. Embryos were incubated at 23 °C until the required stages of embryogenesis stages.

### Western blot analysis

Protein lysates were prepared by homogenizing embryos in lysis buffer (137 mM NaCl, 20 mM Tris–HCl pH 8.0, 1% Nonidet-P40, and 10% glycerol) supplemented with 1 mM phenylmethylsulfonyl fluoride, 5 mM sodium orthovanadate, and 1X protease inhibitor mixture. Embryonic lysates were heated at 95 °C in loading buffer for 5 min and electrophoresed with 12% SDS-PAGE. Western blots were probed with monoclonal anti-Flag (1:1000; Applied Biological Materials, Richmond, Canada) and goat anti-mouse horseradish peroxidase-conjugated antibodies (1:10,000; Santa Cruz Biotechnology, Dallas, TX). The immunoreactive proteins were detected with an enhanced chemiluminescence (ECL) kit (HyClone, Logan, UT).

### Whole-mount in situ hybridization

Two-cell and eight-cell stage embryos were unilaterally injected with *kdm5c* MO and fixed at appropriate stages in MEMFA (4% paraformaldehyde, 0.1 M MOPS pH 7.4, 1 mM MgSO_4_, and 2 mM EGTA) overnight at 4 °C and then dehydrated in 100% methanol prior to storage at − 20 °C. To prepare the antisense digoxigenin-labeled probes, DNA templates were linearized using restriction enzymes. Probes were generated using SP6 or T7 RNA polymerase (Ambion). Probes were detected using alkaline phosphatase-labeled anti-digoxigenin antibodies (1:1000; Roche, Basel, Switzerland) and nitro blue tetrazolium/5-bromo-4-chloro-3-indolyl phosphate [59].

### RT-PCR

Total RNA was extracted from embryos using Isol-RNA lysis reagent (5 Prime GmbH, Hilden, Germany). cDNA was prepared by reverse transcription using a PrimeScript™ first-strand cDNA synthesis kit (Takara, Kusatsu, Japan) with RNA extracted from *Xenopus* embryos ranging from stages 0–40 according to standard protocol. PCR was performed using specific primer pairs (Table 1). PCR products were separated on 1% agarose gels, and images were captured using WiseCapture I-1000 (Daihan Scientific, Wonju, South Korea) and were analyzed by Image J software (National Institutes of Health, Bethesda, MD, USA).

**Table 1:** Primer sequences for RT-PCR analysis

Gene	Forward primer	Reverse primer
*odc*	5′-CAG CTA GCT GTG GTG TGG-3′	5′-CAA CAT GGA AAC TCA CAC C-3′
*sox3*	5′-AGC GCT TTC TCG TGC AGT-3′	5′-TGC CAG CAG GCA AGT AAA-3′
*pax3*	5′-AGG AGG ACA TGG AGC TGG AT-3′	5′-AAC GGG TAA AGG TTC GCT GT-3′
*twist*	5′-CAG GAA GAG TCC AGC TCG C-3′	5′-GTG GCC TGA GCT GTA GTG G-3′
*slug*	5′-CCC CCT CCA CAA TCT GAC AC-3′	5′-GCC ACG GTC TAG AAA AGG CT-3′
*sox8*	5′-TGT CTC CAG CCG GAT CAG A-3′	5′-CAG CCT CCT CCA CAA AGG G-3′
*sox9*	5′-TTG GTG AGC TGA GCA GTG AG-3′	5′-GCT GTT GCT GTT GGT CAC TG-3′
*sox10*	5′-TAT GGT ATG GGC CCA GGC T-3′	5′-GGG TAG GGG GTC CAT GAC T-3′
*otx2*	5′-TTC CTT CGC GGA TTG TGC T-3′	5′-GAC CAG GGT TCT GTG TGG G-3′
*pax6*	5′-CAG AAC AGT CAC AGC GGA GT-3′	5′-TCACTG CCG GGT ACT TGA AC-3′
*rax*	5′-TCC CCT GAT GGC TGA TGG A-3′	5′-GGC ATG GTG GCT GAT CTG T-3′
*epha4*	5′-CCC AGC AGA ATG GCC TGA A-3′	5′-GGC TGG CTC CTT CAC CAA T-3′
*epha2*	5′-GTA CCC ATT GGC CAC TGC T-3′	5′-TGA TGG TGA TGG TGG TGC C-3′
*ephB2*	5′-AGG ACT GCG ATC TCC TGG T-3′	5′-TTT GCT GGG CTC TGA GGT G-3′
*aldh1a2*	5′-GAC TGC TCT TGC GAC CCT T-3′	5′-GCT CCT GCA GTT GGA CCA T-3′
*wnt8a*	5′-GCG GCT GCA GTG ATA ATG C-3′	5′-ACT CTC GTC CCT CTG TCC C-3′

### Alcian blue staining

*Xenopus* embryos were harvested at stage 45, fixed in Bouin’s solution for 2 h at room temperature, and then washed in 70% ethanol containing 0.1% NH_4_OH. Embryos were stained using 0.05% alcian blue 8GX (Sigma-Aldrich, St. Louis, MO) in 5% acetic acid for 2 h at room temperature. The embryos were then washed in 5% acetic acid for 2 h and cleared in 100% methanol, after which they were incubated in 2:1 benzyl benzoate:benzyl alcohol.

### Vibratome sectioning

Fixed embryos were washed with 1X phosphate-buffered saline (PBS), embedded in 3% low-melting agarose in 1X PBS, and sectioned at 100-μm thickness using a vibratome (VT 1000S; Leica, Wetzlar, Germany).

### TUNEL and pH3 staining

To perform TUNEL and pH3 staining, *Xenopus* embryos were fixed in MEMFA (4% paraformaldehyde, 0.1 M MOPS pH 7.4, 1 mM MgSO_4_, and 2 mM EGTA), washed with PBS, and then bleached in a bleach solution (3% H_2_O_2_, 5% formamide, and 5X SSC). For TUNEL assays, bleached embryos were end-labeled using digoxigenin-11-dUTP (Sigma-Aldrich) and TdT (Invitrogen, Carlsbad, CA). Labeled ends were detected with alkaline phosphatase-labeled anti-digoxigenin antibodies (1:1000; Roche) and nitro blue tetrazolium/5-bromo-4-chloro-3-indolyl phosphate. For pH3 staining, bleached embryos were blocked in a blocking solution (1% bovine serum albumin and 5% goat serum in PBS) and probed with anti-histone H3 (1:1000; Abcam, Cambridge, UK) and anti-rabbit IgG AP-linked antibody (1:2000; Santa Cruz Biotechnology). pH3-positive cells were detected by nitro blue tetrazolium/5-bromo-4-chloro-3-indolyl phosphate [39, 60].

### Transcriptome analysis

Total RNA was extracted from each sample, and an RNA sequencing library was constructed using polyA enrichment according to manufacturer’s instructions (Illumina, San Diego, CA). *X. laevis* cDNA sequence reads were mapped from the genome project consortium [61] using BWA (version 0.7.15) to estimate mRNA abundance, and then, differentially expressed (DE) genes were analyzed using edgeR (version 3.20.7). Genes with greater than fourfold change and false discovery rates (FDR) less than 0.01 in exact tests were considered to show significant differential expression. To test overrepresented biological processes in these DE genes, we used Fisher’s test provided by the PANTHER database (released 20171205) with human orthologous genes based on best hits using BLASTp search. Raw data for RNA-seq are available at the NCBI GEO database (accession number GSE117754) [62].

### Statistical analysis

WISH and RT-PCR data were analyzed using ImageJ software (NIH; http://imagej.nih.gov). Results were interpreted by nonparametric, one-tailed Mann–Whitney rank-sum test using GraphPad Prism 7 software (GraphPad Software Inc., La Jolla, CA). *P* values < 0.05 were considered statistically significant.

## Additional files

**Additional file 1: Fig. S1.** Western blot analysis supports the specificity of the *kdm5c* MO. The embryos were microinjected with *kdm5c* wild-type (WT) RNA with or without *kdm5c* MO. To analyze the specificity of the *kdm5c* MO, we microinjected the embryos with kdm5c* RNA with or without *kdm5c* MO. Western blot analysis revealed that no protein expression was detected for embryos injected with *kdm5c* MO/*kdm5c* WT. On the other hand, strong protein expression of Flag-tagged *kdm5c* was observed in embryos injected with kdm5c* alone or together with *kdm5c* MO. WB, western blot.

**Additional file 2: Fig. S2.** Transcriptome analysis revealed that KDM5C is essential for *Xenopus* embryonic development. We performed RNA-seq and analyzed groups of genes that are essential for several biological processes. The downregulation of specific genes by *kdm5c* knockdown indicated that KDM5C plays significant roles in organ development and structure morphogenesis.

## Abbreviations

KDM5C: lysine-specific histone demethylase 5C
KMTs: lysine-specific methyltransferases
DNMTs: DNA methyltransferases
JmjC domain: Jumonji C domain
JmjN domain: Jumonji N domain
PHD: plant homeodomain
XLMR: X-linked mental retardation
siRNA: small interfering RNA
MO: morpholino oligonucleotide
WISH: whole-mount in situ hybridization
RT-PCR: reverse transcription-polymerase chain reaction
ODC: ornithine decarboxylase
FDR: false discovery rate
SOX-E family: SRY-related HMG box containing family of transcription factors
