# Supplementary figures and images for: Physiological effects of KDM5C on neural crest migration and eye formation during vertebrate development
Source: Epigenetics Chromatin. 2018 Dec 6;11:72. doi: 10.1186/s13072-018-0241-x (PMC6282277; doi:10.1186/s13072-018-0241-x)

## Slide 1
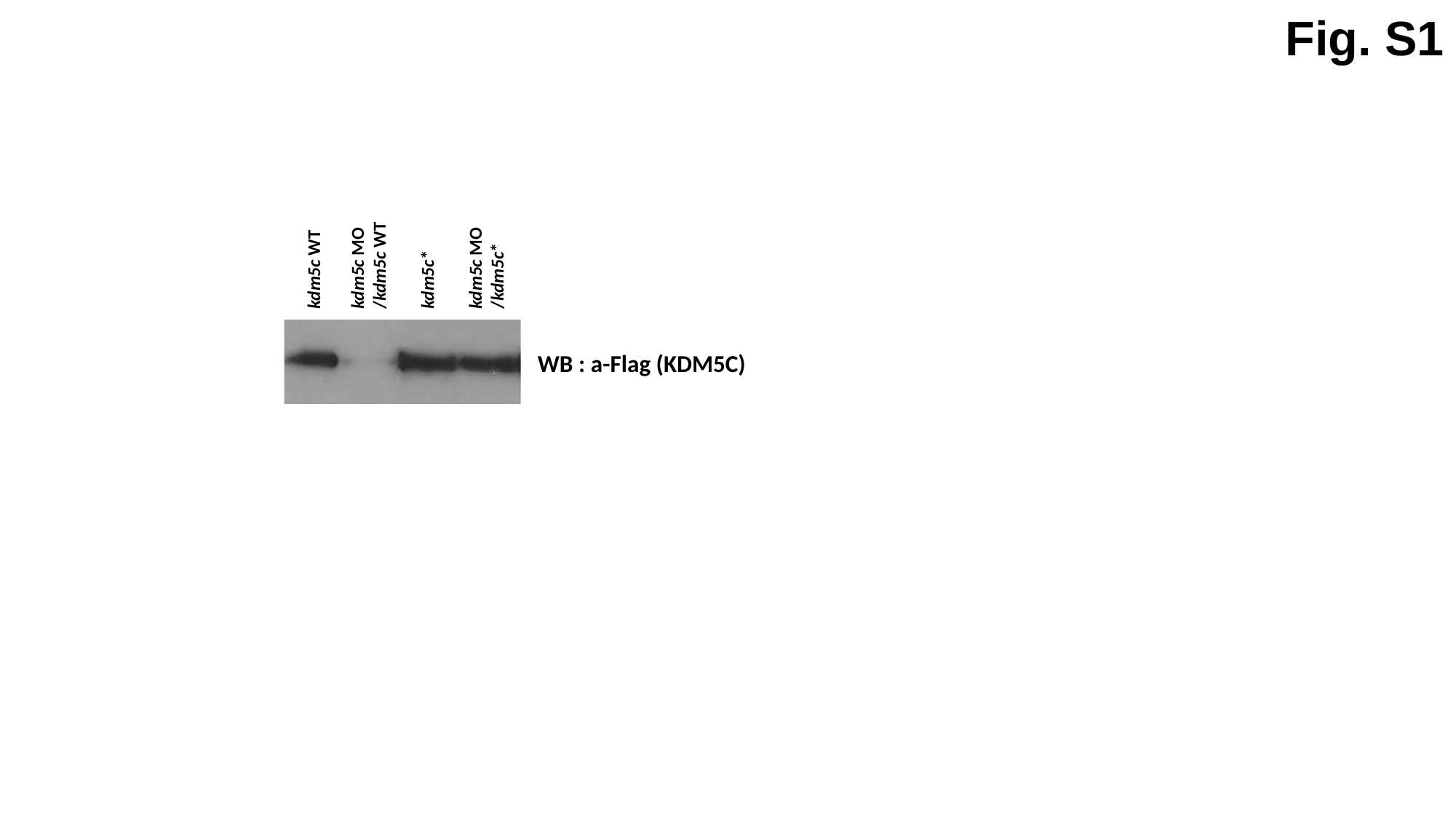

Fig. S1
kdm5c MO
/kdm5c*
kdm5c MO
/kdm5c WT
kdm5c*
kdm5c WT
WB : a-Flag (KDM5C)

Supplement: Supplementary file 1 — Additional file 1: Fig. S1. Western blot analysis supports the specificity of the kdm5c MO. The embryos were microinjected with kdm5c wild-type (WT) RNA with or without kdm5c MO. To analyze the specificity of the kdm5c MO, we microinjected the embryos with kdm5c* RNA with or without kdm5c MO. Western blot analysis revealed that no protein expression was detected for embryos injected with kdm5c MO/kdm5c WT. On the other hand, strong protein expression of Flag-tagged kdm5c was observed in embryos injected with kdm5c* alone or together with kdm5c MO. WB, western blot. [file 13072_2018_241_MOESM1_ESM.pptx]

## Slide 1
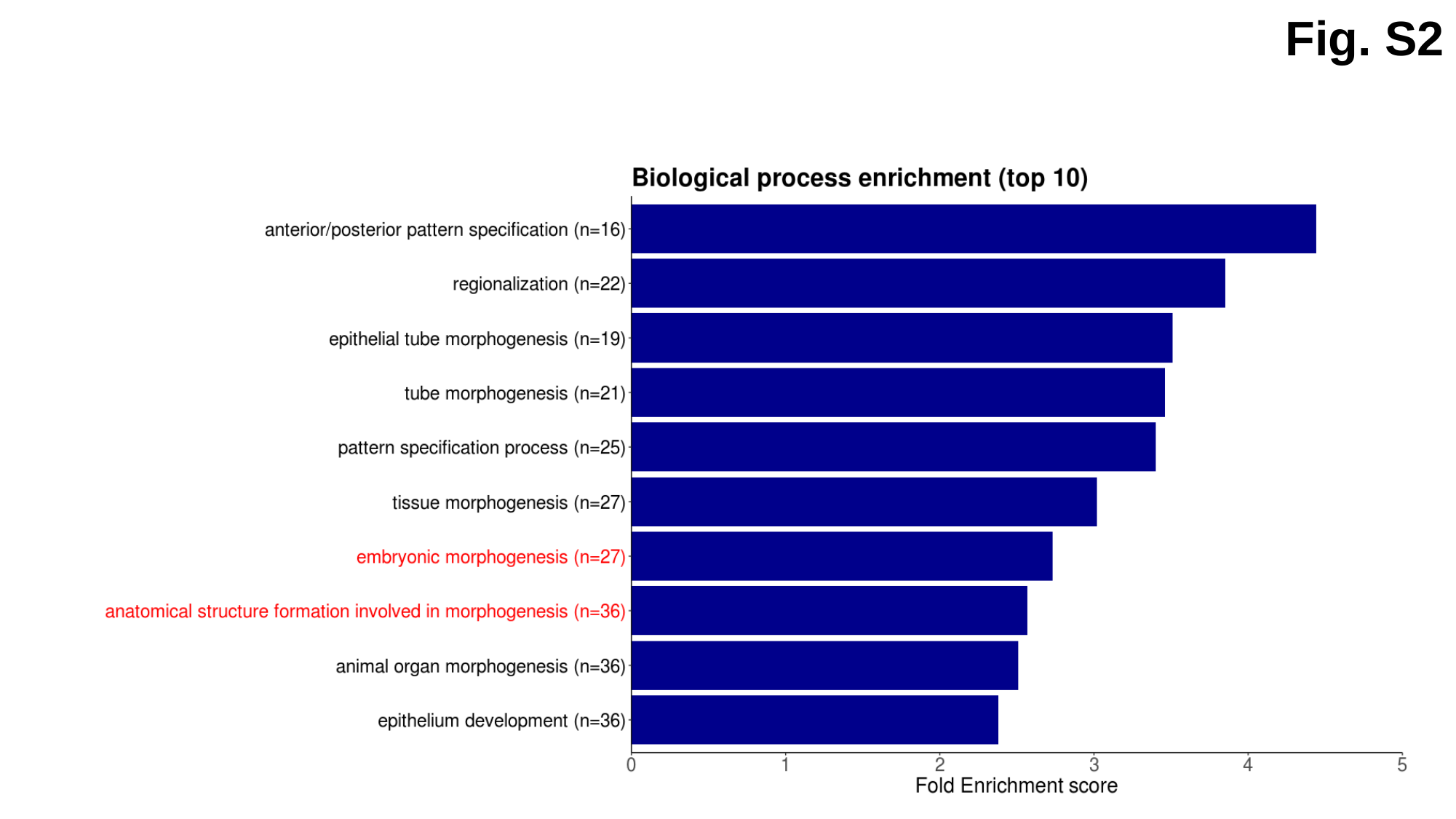

Fig. S2

Supplement: Supplementary file 2 — Additional file 2: Fig. S2. Transcriptome analysis revealed that KDM5C is essential for Xenopus embryonic development. We performed RNA-seq and analyzed groups of genes that are essential for several biological processes. The downregulation of specific genes by kdm5c knockdown indicated that KDM5C plays significant roles in organ development and structure morphogenesis. [file 13072_2018_241_MOESM2_ESM.pptx]
